# Treatment Strategies for Isolated LC-1 Pelvic Injuries: A Comparative Cohort Study of Percutaneous Posterior-Only vs. Combined Anterior–Posterior Fixation

**DOI:** 10.3390/jcm14217507

**Published:** 2025-10-23

**Authors:** Mohammed Rashed Aly Abdelrahman, Frank Hildebrand, Eftychios Bolierakis, Till Berk, Hatem Alabdulrahman

**Affiliations:** 1 Department of Orthopaedics, Trauma and Reconstructive Surgery, RWTH Aachen University, Pauwelsstraße 30, 52074 Aachen, Germany; mabdelrahman@ukaachen.de (M.R.A.A.); ebolierakis@ukaachen.de (E.B.); hrahman@ukaachen.de (H.A.); 2 Department of Orthopaedic Surgery, Zagazig General Hospital, Zagazig 44511, Egypt

**Keywords:** lateral compression type 1, LC1, pelvic ring instability, combined anterior–posterior fixation, posterior-only fixation, percutaneous fixation

## Abstract

**Background:** The management of lateral compression type 1 (LC-1) pelvic fractures remains controversial. Posterior fixation alone has traditionally been practiced without clearly defined indications for supplementary anterior stabilization. Direct comparative evidence between posterior-only and combined anterior–posterior fixation remains scarce. This study evaluated whether institutional criteria reliably identify patients who benefit from additional percutaneous anterior fixation. **Methods:** A retrospective cohort study was conducted at a level I trauma center and included adults with LC-1 fractures treated exclusively by percutaneous fixation. Combined anterior–posterior fixation was performed when predominant anterior pain and radiographic compromise indicated instability. Primary outcomes were pain trajectory (Numeric Rating Scale), inpatient opioid use, physiotherapy clearance, and ward mobility. **Results:** Thirty-seven patients were analyzed (combined = 14; posterior-only = 23). Preoperative pain was higher in the combined group (median 7 vs. 6; median difference 1 [95% CI 0 to 2]; *p* = 0.0036). Postoperatively, pain scores were lower in the combined group at 1–6 weeks (median difference −1 [95% CI −2 to 0]; *p* < 0.05). Opioid consumption was reduced (193 mg vs. 312 mg; median difference −200 mg [95% CI −280 to −120]; *p* < 0.001), and physiotherapy clearance occurred earlier (4 vs. 7 days; median difference −3 [95% CI −5 to −1]; *p* = 0.020). **Conclusion:** Our current indications to perform combined fixation were associated with favorable early outcomes in pain control and physiotherapy clearance among patients with LC-1 fractures showing anterior compromise. These results support a selective combined approach, though interpretation must remain cautious given the small retrospective cohort. Further prospective studies are warranted to validate these findings and refine patient selection.

## 1. Introduction

Pelvic fractures represent a severe trauma entity, accounting for 3% of all fractures [1]. Lateral compression type 1 (LC-1) fractures constitute 50–60% of pelvic ring injuries, representing the most prevalent subtype within this injury spectrum [2]. Historically, LC-1 fractures have been classified as stable injuries within the Young–Burgess classification [3]. Emerging evidence suggests that this classification oversimplifies a wide spectrum of fracture morphologies, clinical variability and biomechanical behaviours, including unstable fracture patterns [4].

Unstable LC-1 fractures show high heterogeneity, with no currently agreed protocol for surgical intervention [5,6]. Prior reports have proposed that posterior-only fixation (P-Fix), which targets the sacrum as the principal fracture component, could be sufficient for restoring pelvic stability in LC-1 injuries [7]. However, pubic rami fractures, forming the anterior element of LC-1 injuries, may significantly hinder early mobilization and often lead to a prolonged and painful recovery [8]. Consequently, combined anterior–posterior fixation (C-Fix) has been proposed to address residual anterior instability, thereby improving early stability, pain control, and functional recovery [9,10].

Existing biomechanical and clinical–radiographic studies have examined the role of anterior fixation in enhancing pelvic ring stability [10,11,12]. However, clinical comparative evidence remains scarce, and available results are inconsistent. Reports have shown improved early stability and reduced analgesic use following combined fixation [9,13], whereas others observed no significant functional advantage [14,15]. These findings emphasize the need for clearer, patient-centered indications for anterior stabilization [16].

This study aimed to compare clinical and functional outcomes between P-Fix and C-Fix in patients with LC-1 injuries managed at our level I trauma centre. Patient selection for anterior fixation followed institutional indications identifying anterior instability through concordant clinical findings and radiographic evidence of anterior compromise. We hypothesized that C-Fix would provide superior early pain relief and faster functional recovery compared with P-Fix in patients exhibiting anterior compromise.

## 2. Methods

### 2.1. Ethical Considerations

This study was approved by the Ethics Committee of the RWTH Aachen Faculty of Medicine (Protocol No. EK 25-332).

### 2.2. Study Design

This retrospective cohort study was conducted at a Level I trauma centre over a five-year period, between 1 January 2019 and 31 January 2024. Data were extracted from electronic medical records and radiological archives. Patients were identified using an administrative database search based on relevant ICD-10 codes related to pelvic fractures.

### 2.3. Inclusion and Exclusion Criteria

Adult patients (aged 18 years or older) with LC-1 fractures, documented in medical records according to the Young–Burgess classification and confirmed by CT, were screened for inclusionPatients were eligible if they underwent surgical treatment exclusively with percutaneous fixation and completed at least six months of clinical and radiological follow-up. Exclusion criteria were defined a priori and comprised polytrauma (Injury Severity Score ≥ 16 or multi-system injuries requiring ICU-level resuscitation), major concomitant musculoskeletal injuries that independently limited postoperative mobilization (e.g., fractures of the lower limb or spine), alternative fixation methods outside the standardized percutaneous protocol (e.g., ORIF or external fixator), and incomplete follow-up or insufficient clinical documentation. To evaluate the potential for selection bias, baseline characteristics of excluded patients were compared with those of the included cohort.

### 2.4. Study Groups

**Figure 1:**
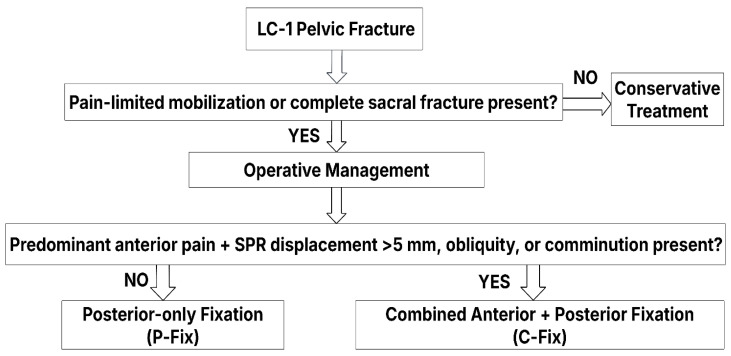
Standard operating protocol (SOP) for management of LC-1 pelvic fractures.

Patients were divided into two groups based on the surgical treatment received: either the posterior-only fixation (P-Fix) group or the combined anterior and posterior fixation (C-Fix) group. Treatment decisions are made at the study location according to a standard operative procedure (SOP) algorithm (Figure 1), considering fracture morphology, displacement, and weight-bearing elicited pain.

Treatment decisions were made by the pelvic trauma team, integrating established treatment guidelines, published indications, and clinical as well as radiological factors to optimize patient outcomes [3,17].

In patients with incomplete sacral fractures diagnosed by CT (mostly of Denis Zone I), a supervised mobilization trial was performed [18]. Non-operative management was selected for those patients who were able to tolerate weight-bearing without significant pelvic pain under adequate analgesia according to the WHO analgesic ladder [19].

Posterior percutaneous fixation was indicated in patients who could not be mobilized due to severe pain or when CT demonstrated a complete sacral fracture [20,21]. Anterior fixation (C-Fix) was added in cases of predominant anterior pain during mobilization, particularly when associated with comminution, oblique fracture lines, or static pubic ramus displacement more than 5 mm [10,11,12,16].

### 2.5. Surgical Procedures

**Figure 2:**
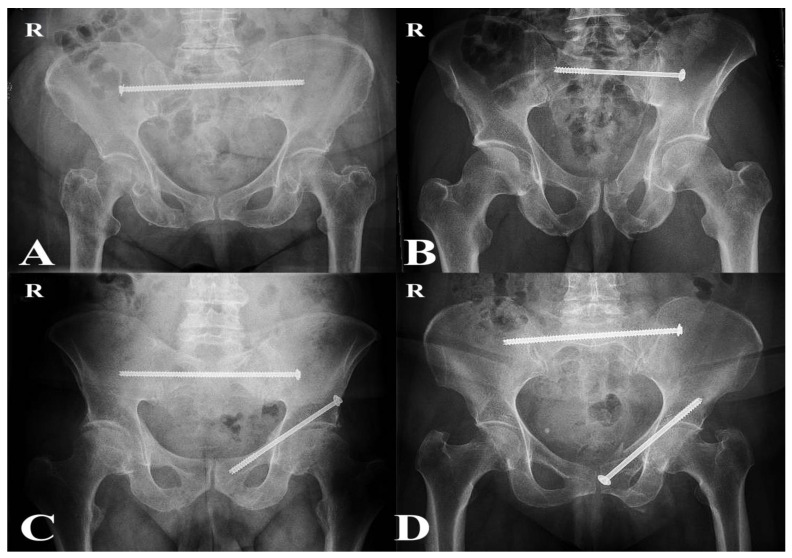
Postoperative radiographs extracted from the hospital’s radiology system (Philips IntelliSpace PACS) illustrating fixation techniques used in both groups. (**A**) Transiliac–transsacral (TITS) screw fixation (P-Fix group); (**B**) Sacroiliac screw (SIS) fixation (P-Fix group); (**C**) Combined TITS and retrograde anterior ramus screw fixation (C-Fix group); (**D**) Combined TITS and antegrade anterior ramus screw fixation (C-Fix group). The letter “R” denotes the right side of the pelvis.

Operations were performed by approximately six senior orthopedic trauma surgeons with subspecialty expertise in pelvic and acetabular surgery. All patients underwent percutaneous fixation under fluoroscopic guidance in the supine position. Trans-iliac trans-sacral (TITS) screws were the preferred technique for posterior fixation. Patients with sacral dysmorphism and incomplete sacral fractures were treated with sacroiliac screws (SIS), which were considered sufficient to provide adequate posterior stability [22]. For anterior fixation, patients received either antegrade or retrograde pubic ramus screws, depending on the individual fracture morphology (Figure 2) [23].

### 2.6. Documented Data

Baseline demographic and clinical variables included gender, age, body mass index (BMI), Charlson Comorbidity Index (CCI), length of hospital stay (LOS) and follow-up duration [24].

### 2.7. Pain Assessment and Analgesic Management

Pain was managed for all patients according to the WHO analgesic ladder to provide adequate pain relief [25]. Preoperative pain levels were assessed using the Numeric Rating Scale (NRS, 0–10), and postoperative pain was evaluated at 1 week, 2 weeks, 6 weeks, 3 months, and 6 months [26].

Pain control medication was monitored throughout the hospital stay. Patients were categorized based on analgesic exposure as receiving opioids only, NSAIDs only, or a combination of both during admission. The use of supportive non-opioid analgesics such as paracetamol and metamizole was also documented.

The total Morphine Milligram Equivalent (MME) administered during hospital admission was calculated for each patient based on established guidelines [27,28]. The calculation followed the standardized formula: Total MME = strength per unit × units per day × number of days × MME conversion factor [29,30,31]. Pain control status at discharge was assessed from final clinical notes, documenting whether the patient was discharged with satisfactory pain control or with residual discomfort.

### 2.8. Radiological Evaluation

Pre- and postoperative imaging was retrieved from the hospital’s radiology system (IntelliSpace PACS). Standard anteroposterior, inlet, and outlet radiographs, as well as CT scans, were evaluated. Sacral fractures were classified according to the Denis classification and further assessed on CT to determine completeness (complete vs. incomplete), with sacral displacement defined as a >5 mm step-off. Superior pubic ramus (SPR) displacement was measured on calibrated AP, inlet, and outlet radiographs. Displacement was defined a priori as >5 mm at the fracture site. Specific fracture morphology, such as oblique SPR fractures, and the zone of involvement according to the Nakatani classification were also evaluated. All radiographic assessments were independently reviewed by two experienced pelvic trauma surgeons according to predefined classification and measurement criteria, demonstrating substantial interobserver agreement.

### 2.9. Functional Evaluation

Postoperative mobilization was initiated on day 1 as tolerated. Patients capable of complying were mobilized with partial weight-bearing, while geriatric patients who were unable to adhere to partial restrictions were mobilized with full weight-bearing. Progressive mobilization was guided by pain levels and radiographic healing.

Functional parameters routinely assessed by the physiotherapy team were retrospectively extracted from medical records for analysis. Key variables included mobility status, weight-bearing capacity, sitting ability, gait independence, use of walking aids, and stair navigation. Mobility condition was monitored using the Surgical ICU Optimal Mobilization Score (SOMS) ranging from 0 (no mobility) to 4 (independent walking) [32].

Mobility status was further evaluated at 3- and 6-month follow-up visits, where patients were categorized into four groups: dependent mobility (wheelchair-bound or bedridden), walking with assistance (e.g., crutches or walker), walking with minimal support, and independent walking without aids or limitations.

### 2.10. Complication Assessment

Surgical complications comprised surgical site infections (SSIs), implant loosening, and revision surgery. Systemic complications included deep vein thrombosis (DVT), pulmonary embolism, urinary tract infection (UTI), and pneumonia.

### 2.11. Statistics

Statistical analysis was performed using SPSS version 28 (IBM Corp., Armonk, NY, USA). Data normality was assessed using the Shapiro–Wilk test. Parametric continuous variables were presented as mean ± standard deviation (SD) and compared using the unpaired t-test. Non-parametric continuous variables were expressed as median and interquartile range (IQR) and compared using the Mann–Whitney U test, with Hodges–Lehmann median differences and corresponding 95% confidence intervals reported to indicate effect size. Categorical variables were expressed as frequencies (%) and compared using the Chi-square or Fisher’s Exact test, as appropriate. All differences were presented with 95% confidence intervals. A two-tailed *p* < 0.05 was considered statistically significant.

## 3. Results

A total of 540 pelvic-ring fractures were screened for eligibility in this retrospective study. Among them, 226 were classified as LC-1 injuries according to the Young–Burgess classification. Of these, 131 patients were managed non-operatively, while 96 underwent surgical fixation. From the surgically treated group, 20 patients were excluded due to polytrauma (*n =* 16) or concomitant major musculoskeletal injuries that limited postoperative mobilization and pain assessment (*n =* 4). All polytrauma injuries resulted from high-energy mechanisms, including motor-vehicle collisions (*n =* 7), falls from height > 3 m (*n =* 6), suicidal jumps (*n =* 3), pedestrian–vehicle impacts (*n =* 2), an industrial crush accident (*n =* 1), and a horse-trampling fall (*n =* 1). The median ISS was 38 (range 28–55), reflecting the extent of multisystem trauma that frequently involved the spine, thorax, abdomen, or lower limbs.

Additionally, 28 patients were excluded because of incomplete or insufficient follow-up data despite meeting the fixation criteria. Another 11 patients were excluded for having received alternative or hybrid fixation techniques outside the standardized percutaneous protocol, including posterior plating (*n =* 3) and combined anterior stabilization using plates (*n =* 3), external fixators (*n =* 3), or INFIX systems (*n =* 2; Medtronic Longitude™ Rod and Screw System). This resulted in a final analytic cohort of 37 patients, all of whom were treated exclusively with percutaneous fixation techniques. Of these, 23 received posterior-only fixation, and 14 underwent combined anterior-posterior percutaneous fixation.

Comparative analysis between included (*n =* 37) and excluded (*n =* 59) patients showed no significant demographic differences. Both cohorts demonstrated similar gender and age distributions, while excluded patients had slightly higher comorbidity scores and longer hospital stays. The follow-up duration was significantly shorter among excluded patients (*p* < 0.001). Fixation patterns were comparable, with posterior-only fixation being the predominant method in both groups (62% vs. 54%). Baseline characteristics of included and excluded patients are summarized in Table 1.

Baseline characteristics in the analyzed cohort were generally balanced between the C-Fix and P-Fix group. The mean age was slightly higher in the C-Fix group (74.1 vs. 67.4 years, *p* = 0.240), and a female predominance was observed in both groups. Also, for all other observed parameters no significant differences were found (Table 2).

Radiographic analysis demonstrated that displacement >5 mm, oblique fracture lines, and comminution were significantly more frequent in the C-Fix group, reflecting the selection of patients with greater anterior instability for combined fixation. All sacral fractures were Denis Zone I, with a similar distribution of complete and incomplete patterns between groups (Table 3). For fixation methods, TITS screws were more commonly used than SIS in both groups, without a statistically significant difference between them. Among patients in the C-Fix group, retrograde screws were the predominant method of anterior fixation.

Postoperative complications were infrequent in both groups. In the C-Fix group, one patient required implant removal due to loosening after fracture consolidation. One urinary tract infection occurred in each group, while pneumonia and pulmonary embolism were each reported in a single patient from the P-Fix group. No cases of surgical site infection or deep vein thrombosis were identified. None of the events required statistical comparison due to their rarity.

Pain outcomes were assessed longitudinally using NRS, revealing a significant benefit of combined C-Fix in the early postoperative period. Preoperatively, patients in the C-Fix group reported higher baseline pain scores than those in the P-Fix group (median NRS 7 vs. 6, *p* = 0.0036). Nonetheless, the C-Fix cohort exhibited superior pain relief across all postoperative timepoints up to 6 weeks. Pain scores continued to improve in both groups beyond this period, with no statistically significant difference at 3 and 6 months (Table 4).

The majority of patients in both groups required opioids, either alone or in combination with NSAIDs, with a higher proportion observed in the P-Fix group (95.7%) compared to the C-Fix group (71.4%). However, this difference did not reach statistical significance (*p* = 0.057). Isolated NSAID use was infrequent and showed no relevant difference between groups. The total inpatient morphine milligram equivalent (MME) was significantly lower in the C-Fix group (193 mg vs. 312 mg, *p* < 0.001), reflecting a reduced opioid requirement. At discharge, most patients in both groups achieved satisfactory pain control, with no significant difference between them. Among the four patients in the P-Fix group with positive anterior radiographic signs, opioid consumption was higher (mean MME 380 mg) when these patients were isolated for analysis. One of them reported irritating groin pain, although this did not necessitate escalation of analgesic therapy. In the C-Fix group, a single patient experienced mild posterior discomfort. Overall, the majority of patients in both groups achieved satisfactory pain control at discharge (Table 5).

Postoperative functional outcomes were generally favourable in both groups, with 100% of patients achieving early mobilization and engaging in physical therapy. Notably, physiotherapy clearance before discharge was achieved significantly earlier among C-Fix patients (4.0 vs. 7.0 days, *p* = 0.020). While most early mobility parameters were similar across groups, a higher proportion of C-Fix patients demonstrated independent or minimally assisted walking (14.3% vs. 4.3%) and completed more than 20 steps (42.9% vs. 21.7%), though these differences were not significant. A small number of patients reached higher functional milestones before discharge: three C-Fix patients (21.4%) and one P-Fix patient (4.3%) were able to walk more than 50 m. Notably, stair navigation capacity differed significantly, with 64.3% of C-Fix patients able to ascend or descend stairs compared to 21.7% in the P-Fix group (*p* = 0.049). All patients in both groups were able to ambulate by discharge, with no observed cases of immobility.

Overall mobility status was comparable between the two groups at both 3 and 6 months. Independent walking was more frequently observed in the C-Fix group at 3 months (35.7% vs. 17.4%), while most patients in both groups walked with minimal support (64.3% vs. 78.3%), though these differences were not statistically significant (*p* = 0.451). At 6 months, 12 patients (85.7%) in the C-Fix group and 18 (78.3%) in the P-Fix group achieved independent ambulation, indicating no significant difference between groups (*p* = 0.656) (Table 6).

## 4. Discussion

Defining which LC-1 fracture patterns require more than posterior fixation remains a major clinical challenge [10,11,12,14,33]. Our results suggest that combining functional and morphological assessment of anterior stability might allow for more precise tailoring of fixation strategies. This approach helps to clarify when C-Fix might be favoured to optimize early recovery. In this context, our study highlights these key findings that may refine surgical decision-making in LC-1 injuries:

Our criteria for anterior stabilization yielded earlier pain relief and reduced opioid requirements, highlighting their clinical relevance.

P-Fix was associated with higher early pain and opioid use despite radiographic anterior stability. As early mobilization is paramount, particularly in elderly patients, these findings suggest that C-Fix may be advantageous in selected cases.

According to our results, percutaneous C-Fix was not associated with additional complications. While late outcomes were comparable, its safety and short-term benefits underscore the importance of early mobilization as a clinical priority.

Non-operative treatment continues to represent the mainstay of management for LC-1 fractures [19,21]. Patients with incomplete sacral fractures, reported in approximately 50–68% of LC-1 injuries, are generally considered suitable for trial mobilization [34]. Those demonstrating sufficient clinical stability are regarded as candidates for conservative treatment [16]. In our screened population, a substantial proportion of patients were managed non-operatively according to this principle. However, our analytic cohort included only patients for whom percutaneous surgical stabilization was both indicated and feasible.

For those patients requiring an operation due to immobilizing pain, methods of surgical management for LC-1 fractures hinge on defining stability [3]. Posterior fixation is widely accepted as the cornerstone of surgical stabilization in LC-1 fractures as it provides approximately 60% of the pelvis’s structural stability [7]. The more debated question is whether supplemental anterior ring fixation confers significant additional benefits in LC-1 injuries, and if so, in which situations it is indicated [6,15]. Sagi et al. proposed that displacement of the pubic ramus or symphysis exceeding 1 cm during examination under anesthesia (EUA) serves as an indication for surgery [35]. However, Hoskins et al. emphasized that the 1 cm threshold is a convention-based surrogate for instability, lacking validation against clinical or functional outcomes [36]. Notably, 25.6% of patients with <1 cm initial displacement later exceeded this threshold. Furthermore, up to 85% of oblique or comminuted rami fractures progressed beyond 1 cm despite appearing minimally displaced initially. [36]. While EUA has been shown to reveal occult instability that may not be evident on standard imaging, fluoroscopic assessment is limited by subjectivity [10,16]. Force application and displacement estimation vary between surgeons and lack clear thresholds for instability [16]. Moreover, the timing of EUA is difficult to define, while resource demands, anesthesia risks and costs constrain its routine use [10]. These limitations emphasize the need for more reproducible data before it can be established as a standard tool in pelvic fracture management [10,16]. Ellis et al. further demonstrated that anterior pelvic ring morphology, particularly comminuted or oblique pubic rami fractures, may be a more decisive determinant of overall stability than the apparent “completeness” of sacral fractures. In such unstable anterior configurations, posterior-only fixation might be inadequate to prevent secondary displacement [10].

While fracture morphology offers guidance, it remains inconclusive in determining instability [5,18]. Beckmann et al. showed that nearly two-thirds of radiographic patterns failed to achieve consistent interobserver agreement. In clinical practice, persistent pain has been regarded as the primary indication for fixation in low-energy pelvic ring injuries [16]. Yet, the contribution of the anterior component of LC-1 fractures as a source of pain and discomfort limiting mobility has not been sufficiently emphasized in prior studies. At our institution, patients with marked anterior pain undergo radiological assessment for signs of anterior instability. Those demonstrating such findings are selected for anterior fixation to promote early recovery and pain relief. In our cohort, radiographic review showed that displacement, obliquity, or comminution were present in nearly all C-Fix cases. This pattern reflects the deliberate selection of patients with both clinical and radiologic signs of anterior instability. A smaller subset of patients in the P-Fix group also demonstrated similar radiographic findings but without permoninat anterior pain. In these cases, stability was judged intraoperatively as sufficient, likely reflecting preserved ligamentous support, and fixation was therefore limited to the posterior ring [4,17]. Closer inspection of the data showed that P-Fix patients with anterior radiographic signs of instability required higher inpatient opioid doses than the overall P-Fix group. Although numbers were small, C-Fix might be beneficial in such patterns, even with limited anterior pain preoperatively. These patients may represent a subgroup that could benefit from additional anterior stabilization, a concept that requires confirmation in larger prospective studies.

In our study, C-Fix showed greater mechanical stability, reflected by a larger early postoperative reduction in NRS pain scores. This benefit also reduced opioid requirements, with C-Fix patients showing lower total MME despite similar access to NSAIDs and supportive analgesics. Villa et al. reported an average inpatient opioid exposure of ~422 MME after pelvic fracture [37]. Although both groups in our cohort remained well below this benchmark, the further reduction with C-Fix suggests a favorable effect in minimizing inpatient opioid burden and long-term opioid-related risks [37]. The published literature provides only limited comparative evidence on the two fixation strategies for isolated LC-1 fractures in the early postoperative phase [9]. Kumaran et al. recently demonstrated that percutaneous C-Fix in LC-1 and LC-2 injuries significantly reduced displacement on stress examination and produced a marked reduction in pain within 24 h [13]. Tucker et al. investigated stress-positive, minimally displaced LC-1 fractures with similar sacral morphology. They reported a trend toward reduced inpatient opioid use and earlier physiotherapy clearance in patients managed with combined fixation. In addition, a significantly greater proportion were cleared by discharge, with a shorter time to ambulate 15 feet [9]. In our cohort, patients in the C-Fix group achieved physiotherapy clearance sooner, with stair navigation at discharge significantly superior compared with P-Fix. Other mobility-related outcomes, including walking distance and step count, also trended in favour of C-Fix, although these did not reach statistical significance. These findings suggest that adding anterior ring fixation may enhance early recovery in LC-1 injuries, even when preoperative radiographs suggest anterior stability.

In previous reports, similar outcomes have been documented between the two fixation strategies within one year after surgery [14,15]. Petryla et al., in a retrospective series of B2 lateral compression fractures, found no significant difference in one-year functional or quality-of-life outcomes. Importantly, treatment allocation was surgeon-dependent and anterior fracture morphology was not analysed. Moussa et al. similarly reported equivalent results; however, their trial was confined to Tile B and C injuries and did not assess fracture morphology or subgroup outcomes within LC-1 specifically. Aggarwal et al., in a posterior-only series including nine LC-1 cases, described consistently good functional outcomes with universal union and a mean time to unassisted weight bearing of 7.6 weeks, but without characterization of anterior ring morphology [7]. The apparent equivalence between P-Fix and C-Fix reported in previous studies likely reflects the pooling of anteriorly stable and unstable fractures, which obscures the true benefit of selective anterior stabilization. However, this equivalence cannot be assumed in anteriorly unstable variants with only posterior fixation, where C-Fix might help reduce the risk of persistent pain, residual deformity, or non-union [11,12,38]. At six months, mobility parameters were comparable between groups. This supports the possibility that anterior fixation contributes to stable outcomes in anteriorly unstable LC-1 fractures treated with C-Fix.

In our study, we restricted the analysis to percutaneous fixation. Plating and external fixation follow different surgical concepts with distinct pain profiles and rehabilitation demands, making direct comparisons less reliable. Previous comparative studies limited to percutaneous techniques have reported low rates of local complications but did not systematically assess general complications [9,14]. In our series, the overall complication rate was low and did not differ between the two groups. This favorable safety profile indicates that percutaneous C-Fix appears to be a viable option in cases with doubtful anterior stability. In such situations, achieving absolute stability remains consistent with the principles of Enhanced Recovery After Surgery (ERAS), particularly in elderly patients [39]. Given the small sample size and retrospective design, larger prospective trials stratified by fracture pattern and initial displacement are required to define clear indications for anterior fixation. The prospective multicenter AO TOP study, currently ongoing, compares P-Fix with C-Fix and is expected to provide important guidance [40].

## 5. Study Limitations

This study has several limitations. Its retrospective, non-randomized design inherently limits causal inference and introduces a risk of selection bias that cannot be fully excluded. Patients with more complex anterior fracture patterns were more often allocated to combined fixation, which may have contributed to the observed early advantages and reduces strict comparability between groups. The relatively small sample size reduces statistical power and increases the likelihood of both type I and type II errors. Therefore, even statistically significant differences should be interpreted with caution. Although analgesia was prescribed according to the WHO analgesic ladder, variation in individual prescribing practices remained possible. Other potential confounders, including pre-injury mobility status and osteoporosis severity, were not controlled for and could have influenced recovery. Furthermore, the six-month follow-up provides valuable insight into early outcomes but does not permit conclusions regarding long-term function or implant durability. Finally, although several surgeons were involved in the operative care, all were senior pelvic specialists working under a standardized institutional protocol, which minimizes but cannot entirely eliminate inter-surgeon variability.

## 6. Conclusions

In LC-1 fractures with anterior compromise, C-Fix provided earlier pain relief and facilitated improved early mobilization, showing more favorable short-term outcomes than P-Fix in patients without clinical or radiological signs of anterior instability. The favorable safety profile and functional recovery observed with percutaneous combined fixation support its use as a viable option in selected cases where anterior stability is uncertain. However, given the retrospective design and limited sample size, these findings should be interpreted with caution and validated through larger prospective studies to establish clear indications for anterior fixation in LC-1 injuries.

## Figures and Tables

**Table 1:** Comparative baseline characteristics of included vs. excluded LC-1 patient cohorts.

Variable	Included (*n =* 37)	Excluded (*n =* 59)	Difference (95% CI)	*p*-Value ^a^
Age (years) ^b^	70 ± 15	69 ± 16	1 (−6 to 8)	0.682
Gender				
Male (%)	11 (30%)	15 (25%)	+5 (−13 to 23)	0.651
Female (%)	26 (70%)	44 (75%)	−5 (−23 to 13)	0.651
CCI ^c^	2 (2–4)	3 (2–5)	1 (0 to 2)	0.287
LOS (days) ^c^	12 (8–22)	17 (4–58)	−5 (−11 to 1)	0.084
Follow-up period (weeks) ^b^	31.8 ± 14.9	20.1 ± 12.3	11.7 (5.1 to 18.3)	**<0.001**
Fixation approach				
P-Fix (%)	23 (62%)	32 (54%)	+8 (−12 to 28)	0.491
C-Fix (%)	14 (38%)	27 (46%)	−8 (−28 to 12)	0.491

Data are shown as mean ± SD (^b^) for parametric and median (IQR) (^c^) for non-parametric variables, or as percentages (%). *p*-values (^a^) were calculated using the *t*-test, Mann–Whitney U test, or Fisher’s Exact Test, as appropriate. All differences are presented with 95% CIs. Bold values indicate statistically significant results (*p* < 0.05).

**Table 2:** Baseline characteristics of patients treated with C-Fix or P-Fix.

Variable	C-Fix (*n =* 14)	P-Fix (*n =* 23)	Difference (95% CI)	*p*-Value ^a^
Age (years) ^b^	74 ± 14	67 ± 16	6.67 (−3.7 to 17)	0.240
Gender				
Male (%)	2 (14%)	6 (30.0%)	−15.7% (−44.8 to 13)	0.422
Female (%)	12 (86%)	14 (70.0%)	15.7% (−13.3 to 44.8)	0.422
BMI (kg/m^2^) ^b^	25.80 ± 3.87	25.37 ± 2.97	0.43 (−2 to 2.9)	0.725
CCI ^c^	3 (2–4)	2 (2–5)	1 (0 to 2)	0.607
TTS (days) ^c^	6.0 (3–11)	5.0 (4–8)	1 (−1 to 2)	0.958
Follow-up period (weeks) ^b^	29.2 ± 11.1	31.8 ± 14.9	−2.6 (−11.34 to 6.14)	0.467

Data are shown as mean ± SD (^b^) for parametric and median (IQR) (^c^) for non-parametric variables, or as percentages (%). *p*-values (^a^) were calculated using the t-test, Mann–Whitney U test, or Fisher’s Exact Test, as appropriate. All differences are presented with 95% CIs. A *p* < 0.05 was considered statistically significant.

**Table 3:** Fracture displacement, morphology, and surgical fixation methods.

Variable	C-Fix (*n =* 14)	P-Fix (*n =* 23)	Difference (95% CI)	*p*-Value ^a^
≥1 sign of anterior instability (SPR displacement > 5 mm, oblique fracture, or comminution)	14 (100%)	4 (17.4%)	82.6% (63.1 to 94.2)	**<0.001**
Nakatani classification				
Zone I	2 (14.3%)	3 (13.0%)	1.3% (–27.0 to 29.6)	<0.999
Zone II	8 (57.1%)	17 (73.9%)	–16.8% (–48.2 to 14.5)	0.487
Zone III	4 (28.6%)	3 (13.0%)	15.6% (–13.1 to 44.2)	0.390
Osteoporosis	3 (21.4%)	5 (21.7%)	–0.3% (–32.6 to 31.9)	<0.999
Complete sacral fracture	5 (35.7%)	8 (34.8%)	0.9% (–31.0 to 32.8)	<0.999
Incomplete sacral fracture	9 (64.3%)	15 (65.2%)	–0.9% (–32.8 to 31.0)	<0.999
Denis classification (Zone I)	14 (100%)	23 (100%)	—	—
Posterior fixation: SIS	5 (35.7%)	8 (34.8%)	0.9% (–31.0 to 32.8)	<0.999
Posterior fixation: TITS	9 (64.3%)	15 (65.2%)	–0.9% (–32.8 to 31.0)	<0.999
Anterior fixation: Retrograde screw	11 (78.6%)	—	—	—
Anterior fixation: Antegrade screw	3 (21.4%)	—	—	—

Data are shown as frequencies (%). *p*-values (^a^) were calculated using the Chi-square or Fisher’s Exact Test, as appropriate. All proportional differences are presented with 95% CIs. Bold values indicate statistically significant results (*p* < 0.05). Anterior fixation was exclusive to the C-Fix group; no statistical comparison was performed.

**Table 4:** Pre- and postoperative pain levels (NRS) in the C-Fix and P-Fix groups.

Timepoint	C-Fix (*n =* 14)	P-Fix (*n =* 23)	Difference (95% CI)	*p*-Value ^a^
Pre-op	7 (6–8)	6 (6–7)	1 (0 to 2)	**0.0036**
1st week	4 (3–5)	5 (4–6)	–1 (–2 to 0)	**0.0076**
2nd week	3 (2–4)	4 (3–5)	–1 (–2 to 0)	**0.0107**
6 weeks	2 (1–3)	3 (2–4)	–1 (–2 to 0)	**0.0016**
3 months	2 (1–2)	2 (2–3)	0 (–1 to 1)	0.089
6 months	1 (0–2)	1 (1–2)	0 (–1 to 0)	0.105

Data are shown as median (IQR) based on the Numeric Rating Scale (NRS), where 0 = no pain and 10 = worst pain. *p*-values (^a^) were calculated using the Mann–Whitney U test. Differences are expressed as Hodges–Lehmann median differences with 95% CIs; a 95% CI including 0 indicates no statistical significance. Bold values indicate statistically significant results (*p* < 0.05).

**Table 5:** Analgesic use and pain control during inpatient stay.

Variable	C-Fix (*n =* 14)	P-Fix (*n =* 23)	Difference (95% CI)	*p*-Value ^a^
Opioid use (%)	10 (71.4%)	22 (95.7%)	−24.3 (−53.6 to 5.0)	0.057
Supportive analgesic use (paracetamol or metamizole) (%)	14 (100%)	21 (91.3%)	8.7 (−6.3 to 23.8)	0.517
Total inpatient MME (mg) ^c^	193 (120–240)	312 (260–390)	−200 (−280 to −120)	**<0.001**
Satisfactory pain control (%)	13 (92.9%)	22 (95.7%)	−2.8 (−22.9 to 17.3)	<0.999

Data are shown as median (IQR) (^c^) for non-parametric continuous variables and as frequencies (%) for categorical variables. *p*-values (^a^) were calculated using the Mann–Whitney U or Fisher’s Exact Test, as appropriate. Differences are expressed as Hodges–Lehmann median or proportional differences with 95% CIs. Bold values indicate statistically significant results (*p* < 0.05).

**Table 6:** Postoperative mobility and functional recovery during hospitalization and at follow-up.

Variable	C-Fix (*n =* 14)	P-Fix (*n =* 23)	Difference (95% CI)	*p*-Value ^a^
**In-hospital course**				
LOS (days) ^c^	11 (8–19)	13 (10–23)	–2 (–7 to 1)	0.080
Physiotherapy clearance (days) ^c^	4 (3–6)	7 (5–9)	–3 (–5 to –1)	**0.020**
Standing with support (%)	12 (85.7%)	22 (95.7%)	–10.0 (–27.8 to 7.8)	0.338
Partial weight-bearing (%)	12 (85.7%)	21 (91.3%)	–5.6 (–24.4 to 13.2)	0.647
Cane as main gait aid (%)	12 (85.7%)	22 (95.7%)	–10.0 (–27.8 to 7.8)	0.338
Distance walked 10–50 m (%)	11 (78.6%)	22 (95.7%)	–17.1 (–40.6 to 6.4)	0.161
Number of steps 1–20 (%)	8 (57.1%)	18 (78.3%)	–21.2 (–53.1 to 10.7)	0.288
Stairs—able to ascend/descend (%)	9 (64.3%)	5 (21.7%)	42.6 (10.4 to 74.8)	**0.049**
**Follow-up mobility**				
Walking with minimal support—3 months (%)	9 (64.3%)	18 (78.3%)	–14.0 (–44.3 to 16.3)	0.451
Independent walking—6 months (%)	12 (85.7%)	18 (78.3%)	7.4 (–20.2 to 35.0)	0.656

Data are shown as median (IQR) (^c^) for non-parametric continuous variables and as frequencies (%) for categorical variables. *p*-values (^a^) were calculated using the Mann–Whitney U or Fisher’s Exact Test, as appropriate. Differences are expressed as Hodges–Lehmann median or proportional differences with 95% CIs. Bold values indicate statistically significant results (*p* < 0.05).

## Data Availability

The collected data will be stored securely in our institute for 10 years. After 10 years, the data will be deleted. However, all the datasets analyzed or generated during this study will be available from the corresponding author upon reasonable request.

## Abbreviations

BMI	Body Mass Index
C-Fix	Combined Anterior and Posterior Fixation
CCI	Charlson Comorbidity Index
ERAS	Enhanced Recovery After Surgery
EUA	Examination under anesthesia
INFIX	Subcutaneous Anterior Internal Fixator
ISS	Injury Severity Score
LC-1	Lateral Compression Type 1
LOS	Length of Stay
MME	Morphine Milligram Equivalent
NRS	Numeric Rating Scale
NSAIDs	Nonsteroidal Anti-inflammatory Drugs
P-Fix	Posterior-Only Fixation
SIS	Sacroiliac Screw
SOMS	Surgical ICU Optimal Mobilization Score
SPR	Superior Pubic Ramus
SSI	Surgical Site Infection
TITS	Transiliac–Transsacral Screw
TTS	Time to Surgery
WHO	World Health Organization
